# 2-methoxyestradiol sensitizes breast cancer cells to taxanes by targeting centrosomes

**DOI:** 10.18632/oncotarget.27810

**Published:** 2020-12-01

**Authors:** Randa El-Zein, Jose Thaiparambil, Sherif Z. Abdel-Rahman

**Affiliations:** ^1^Houston Methodist Cancer Center, Houston, TX 77030, USA; ^2^Department of Radiology, Houston Methodist Research Institute, Houston, TX 77555, USA; ^3^Department of Obstetrics and Gynecology, Maternal-Fetal Pharmacology and Biodevelopment Laboratories, The University of Texas Medical Branch at Galveston, Galveston, TX 77555, USA

**Keywords:** breast cancer, 2-methoxyestradiol (2-ME), centrosome amplification, centrosome declustering

## Abstract

Centrosomes amplification is a hallmark of cancer. We hypothesize that 2-methoxyestradiol (2-ME) sensitizes breast cancer (BC) cells to taxanes by targeting amplified centrosomes. We assessed the extent by which 2-ME together with paclitaxel (PTX) induces centrosome alterations with subsequent mitotic catastrophe in different BC subtypes. 2-ME induced a significant reduction in PTX IC_50_ values in all cells tested ranging from 28–44% (*P* < 0.05). Treatment with both PTX and 2-ME significantly increased the number of misaligned metaphases compared to PTX alone (34%, 100% and 52% for MCF7, MDA-MB231 and SUM149, respectively; *P* < 0.05). The number of cells with multipolar spindle formation was significantly increased (81%, 220% and 285% for MCF7, MDA-MB231 and SUM 149, respectively; *P* < 0.05). PTX and 2-ME treatment significantly increased interphase declustering in cancer cells (56% for MCF7, 208% for MDA-MB231 and 218% for SUM149, respectively; *P* < 0.05) and metaphase declustering (1.4-fold, 1.56-fold and 2.48-fold increase for MCF7, MDA-MB231 and SUM149, respectively; *P* < 0.05). This report is the first to document centrosome declustering as a mechanism of action of 2-ME and provides a potential approach for reducing taxane toxicity in cancer treated patients.

## INTRODUCTION

Despite recent advances in early detection and treatment, breast cancer (BC) remains a major public health problem worldwide. In the US, BC is the most common cancer among women, accounting for 30% of newly diagnosed cancers. In 2019, over 268,600 new cases were diagnosed, resulting in more than 41,760 deaths [1]. The economic burden of breast cancer is tremendous, amounting to $18.1 billion yearly [2].

BC is a heterogeneous disease with an array of tumor types classified according to their histological and molecular profiles. Currently, there are at least 4 subtypes namely: Luminal A (ER+, PR+/−, HER2-); Luminal B (ER+, PR+/−, HER2+); Basal (ER-, PR-, HER2-); and HER2+ amplified. Luminal A subtype is responsive to hormone therapy and often to chemotherapy. Luminal B is usualy responsive to hormone and chemotherapy with variable response to HER2 antibody therapies. The Basal type is not repsonsive to hormone therapy but is often responsive to chemotherapy, while the HER2 amplified is often reponsive to HER2 antibody and chemotherapy [3]. The most commonly used chemotherapeutics for treatment of BC are taxanes. Taxane therapy is well established in adjuvant and neoadjuvant management of early stage BC in combination with trastuzumab (Herceptin^®^) and anthracyclines [4–6], and in metastatic BC with success rates ranging from 25%–69% [5, 6]. It is reported that taxanes act mainly through: a) interference with microtubule polymerization, leading to cell cycle arrest at mitosis [7]; b) phosphorylation and inactivation of the anti-apoptotic Bcl-2 protein, leading to the induction of caspase-dependent apoptosis [8]. Since taxane usefulness is limited due to their toxic side effects and *de-novo* refractoriness (acquired tumor resistance), it is imperative to develop better treatment strategies to improve patients response and prognosis to these therapeutics.

Although initiation and progression of BC involve a complex interplay of many environmental and genetic factors, estrogens (E2) play a central role [9]. E2 metabolites could either stimulate or inhibit breast cells growth through diverse mechanisms and can thus impact BC response to treatments [10, 11]. For example, the E2 metabolite 4-hydroxyestradiol (4-OHE) was reported to enhance proliferation of BC cells [10] and to form adducts with taxanes, thus inhibiting their therapeutic effect [12]. 4-OHE can also undergo redox cycling, forming reactive quinones and semiquinones, thereby contributing to hormonal carcinogenesis [13]. On the other hand, another E2 metabolite, 2-methoxyestradiol (2-ME), is reported to possess antiproliferative properties in both estrogen receptor positive (ER^+^) and negative (ER^-^) BC cells [14] and to enhance the therapeutic effects of taxanes [11]. Several mechanisms for the beneficial effect of 2-ME were investigated, among which interference with microtubule polymerization, antiangiogenic and pro-apoptotic actions [15, 16].

Proper functioning of mitotic spindle apparatus is essential for development and cell fate by ensuring accurate distribution of genetic material and normal division plane. The centrosome duplicates during the S-phase to yield two centrosomes that instruct the formation of the bipolar spindle. Formation and correct positioning of a bipolar mitotic spindle is critical for correct chromosome alignment and subsequent segregation [17]. Alterations in the regulatory mechanisms that govern centrosome duplication result in centrosome amplification, which could lead to aberrant mitoses and chromosome segregation errors [18]. Centrosome amplification has been reported in solid and hematological cancers [19, 20] as well as in early and late stages of tumorigenesis and is associated with poor prognosis [21]. Supernumerary centrosomes in cancer cells cause spindle multipolarity which results in a non-viable progeny. The cancer cells can overcome this by clustering the centrosome to assemble a pseudo-bipolar mitotic spindle which can therefore yield viable daughter cells [20]. Therefore, disrupting this clustering would selectively drive the cell with amplified centrosomes (i.e., the cancer cell) to a mitotic catastrophe and apoptosis while at the same time sparing the normal cells.

We tested the hypothesis that 2-ME sensitizes BC cells to taxanes by targeting amplified centrosomes in tumor cells. Our rationale is that both taxanes and 2-ME interfere with microtubule polymerization preventing appropriate function of the spindle apparatus and thus causing cell cycle arrest. We used breast cancer cell lines representing different BC subtypes to evaluate the extent of centrosome amplification and spindle apparatus alternations. We then assessed the extent by which 2-ME in combination with the taxane paclitaxel (PTX) induces centrosome amplification, clustering and declustering with subsequent mitotic catastrophe. Our data document centrosome declustering as a novel mechanism of action of 2-ME and provide a potential approach for reducing taxane toxicity in cancer treated patients.

## RESULTS

### Determination of PTX concentration-response IC_50_ profiles in presence and absence of 2-ME in HR+ and TNBC cell lines

**Figure 1:**
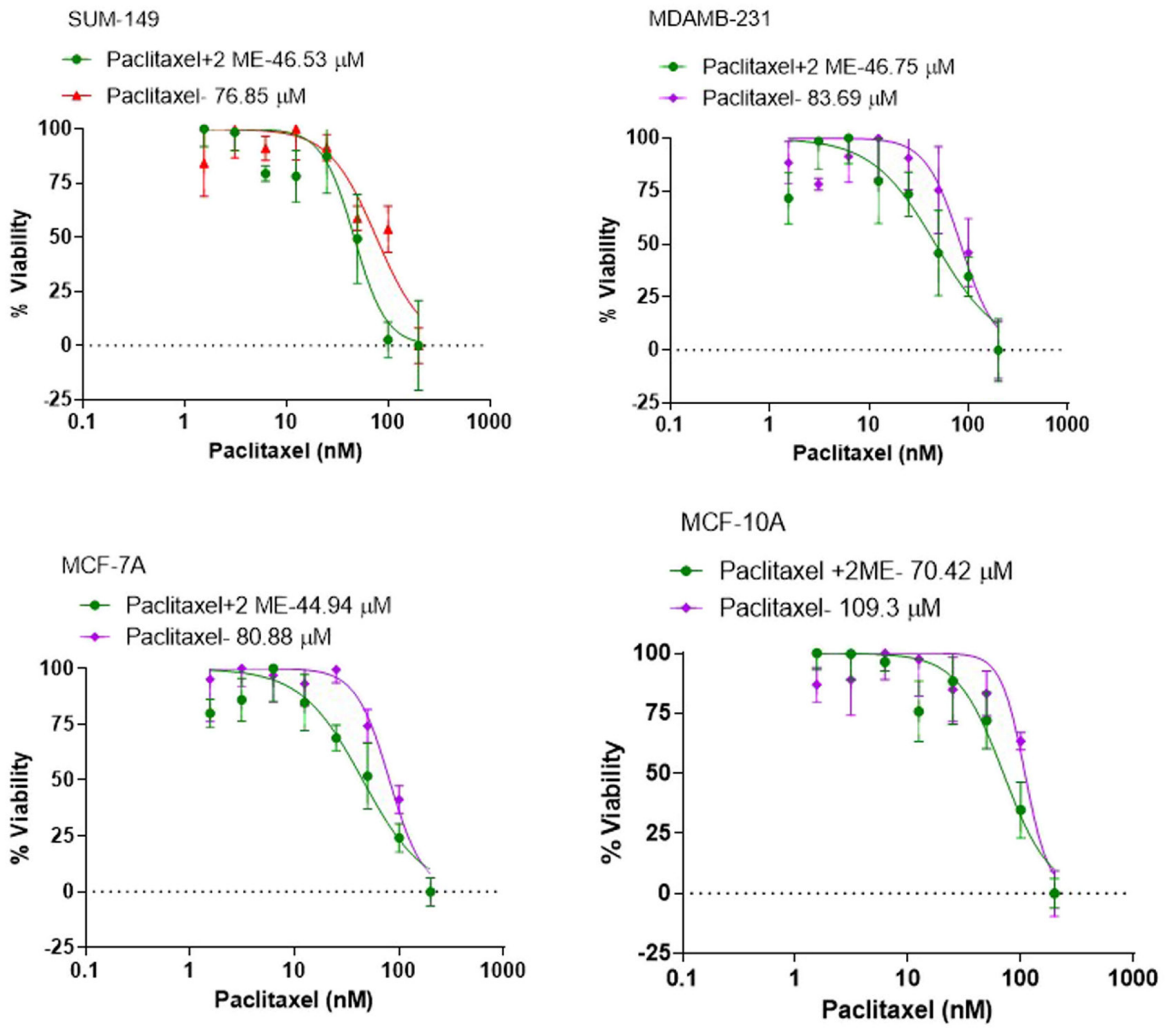
Cytotoxicity studies to determine the IC_50_ of PTX in the different breast cancer cell lines. The IC_50_ for MCF10A cells was 97.96 nM, for MCF7 80.88 nM, for MDA-MB-231 73.87 nM and for SUM 149 cells 76.85 nM. Treatment with 2-ME resulted in a significant reduction in IC_50_ values in all cell lines with 28%, 44%, 37% and 39% reduction for MCF-10A, MCF-7, MDA-MB-231 and SUM 149 respectively.

For proof of concept of our hypothesis that 2-ME sensitizes BC cells to taxanes, we determined the IC_50_ of PTX in 3 different breast cancer cell lines namely: MCF7, MDA-MB- 231, SUM 149 and the non-cancer MCF10A breast cell line as the control. PTX was dissolved in DMSO and drug concentration-effect curves were assessed. Cytotoxicity was assessed at the end of drug exposure using the XTT assay. The IC_50_ for MCF10A cells was 97.96 nM, for MCF7 80.88 nM, for MDA-MB-231 73.87 nM and for SUM 149 cells 76.85 nM. Treatment with 2-ME resulted in a significant reduction in IC_50_ values in all cell lines with 28%, 44%, 37% and 39% reduction for MCF-10A, MCF-7, MDA-MB-231 and SUM 149 respectively (*p* < 0.05) (Figure 1), indicating a higher taxane potency in presence of 2-ME. Based on our cytotoxicity data, we selected the 50 nM concentration of PTX for subsequent experiments.

### Misaligned metaphases and multipolar spindles

**Figure 2:**
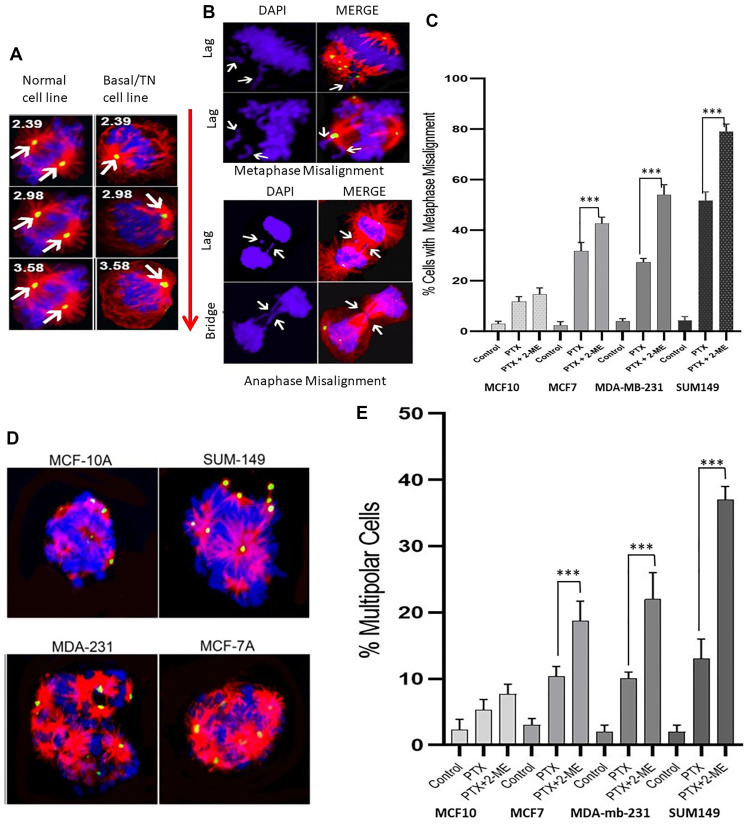
Immunofluorescence images with pericentrin (green), tubulin (red) and DNA (DAPI-blue) staining. (**A**) Confocal planar z sections of tubulin with bipolar spindle oriented parallel to the substratum, and spindle poles in similar z planes in normal and in basal (TN) BC cells. Arrows in left panel (normal cells) indicate spindle poles at opposite sides of the cell. Right panel, basal (TN) cells show bipolar spindles that appear misoriented relative to the substratum, whereby spindle poles were in drastically different confocal z planes compared to those in normal cells at the same confocal plane. Numbers indicate the *z* distance from the top. (**B**) Consequence of misoriented spindle with the generation of misaligned chromosomes at metaphase as well as lagging chromosomes and formation of bridges in anaphase. (**C**) Treatment with both PTX and 2-ME leads to a significant increase in the number of misaligned metaphases compared to treatment with PTX alone (34%, 100% and 52% increases for MCF7, MDA-MB231 and SUM149, respectively). (**D**) and (**E**) The number of cells with multipolar spindle formation was significantly higher in cells treated with both PTX and 2-ME as compared to those treated with PTX alone (81%, 220% and 285% increases for MCF7, MDA-MB231 and SUM 149, respectively).

Spindle tubule misorientation can lead to aberrant chromosome alignment, resulting in cell cycle arrest and abnormal metaphases. Figure 2A is a representative IF image (analyzed using confocal planar z sections of tubulin) with bipolar spindle oriented parallel to the substratum, and spindle poles in similar z planes in normal and in basal (TN) BC cells. Arrows in Figure 2A left panel (normal cells) indicate spindle poles at opposite sides of the cell. In contrast, basal (TN) cells (Figure 2A, right panel) show bipolar spindles that appear misoriented relative to the substratum, whereby spindle poles were in drastically different confocal z planes compared to those in normal cells at the same confocal plane. Numbers indicate the z distance from the top. Figure 2B shows the consequence of misoriented spindle with the generation of misaligned chromosomes at metaphase as well as lagging chromosomes and formation of bridges in anaphase.

Our data (Figure 2C) indicate that treatment with both PTX and 2-ME resulted in a significant increase (*P* < 0.05) in the number of misaligned metaphases compared to treatment with PTX alone (34%, 100% and 52% increases for MCF7, MDA-MB231 and SUM149, respectively). In contrast, no significant difference was observed in MCF10A cells. Similarly, the number of cells with multipolar spindle formation (Figure 2D and 2E) was significantly higher (*P* < 0.05) in cells treated with both PTX and 2-ME as compared to those treated with PTX alone (81%, 220% and 285% increases for MCF7, MDA-MB231 and SUM 149, respectively). No significant difference was observed in MCF10A cells. All these mitotic defects increase chromosome missegregation and lead to cell cycle arrest in breast cancer cells.

### Centrosome amplification and clustering

**Figure 3:**
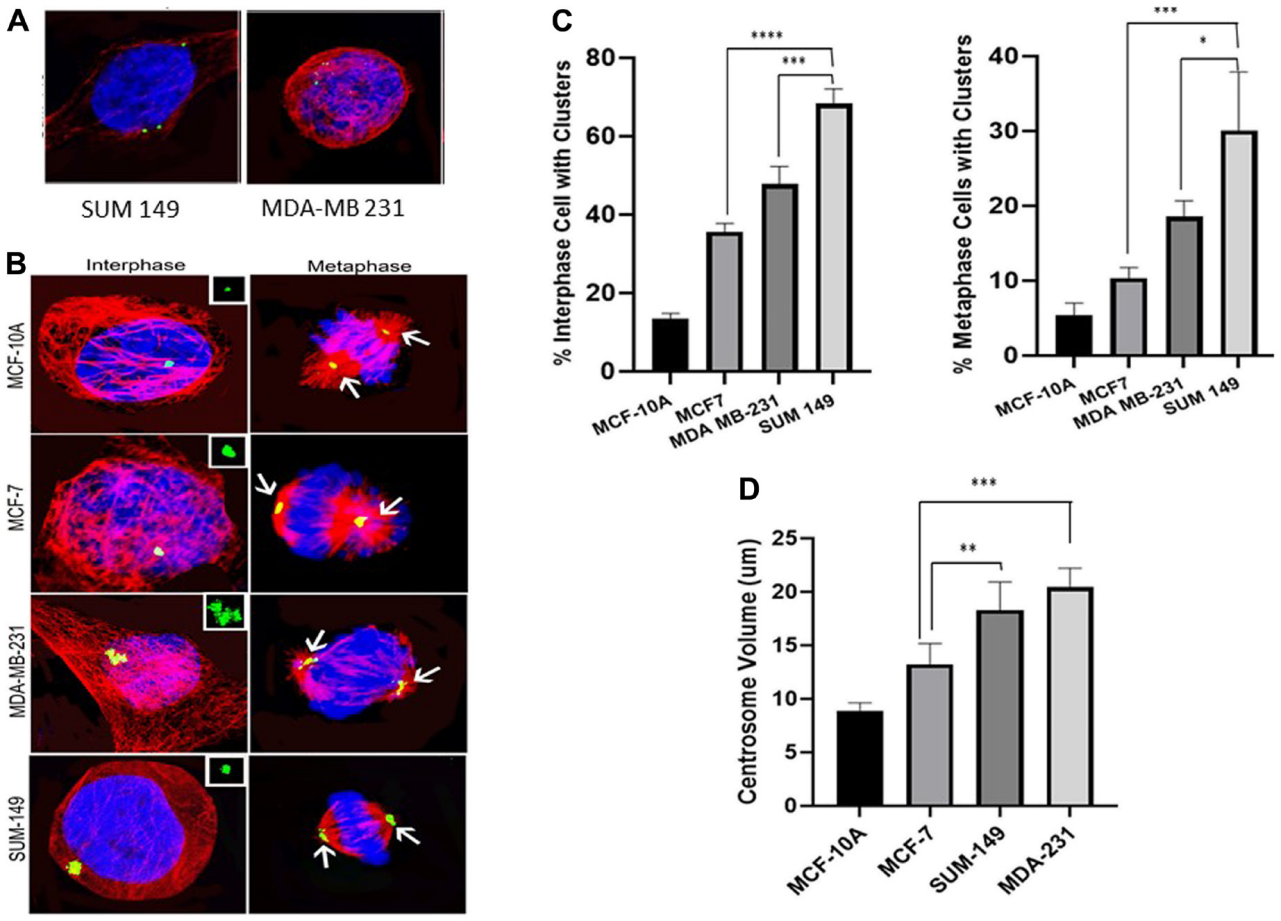
(**A**) Cells with amplified centrosomes (>2 centrosomes). (**B**) Clustered centrosomes in interphase and metaphase cells. (**C**) Extent of centrosome clustering in untreated cells from each of the 4 cell lines. Interphase clustering was significantly higher in MCF7, MDA-MB 231 and SUM 149 as compared to MCF-10A. A significant increase in interphase clustering observed between MDA-MB 231 and MCF7 and between SUM 149 and MCF7. Similarly, SUM 149 interphase clustering was significantly higher than MDA-MB 21 clustering. Metaphase clustering was also significantly higher in MCF7, MDA-MB 231 and SUM 149 as compared to MCF-10A. A significant increase in metaphase clustering was observed between MDA-MB 231 and MCF7 and SUM 149 and MCF7. In addition, SUM 149 metaphase clustering was significantly higher than that observed in MDA-MB 231. (**D**) shows the differences in centrosome volumes in the different cell lines. Centrosome volume was significantly higher in MCF7, MDA-MB 231 and SUM 149 as compared to MCF-10A. A significant increase in centrosome volumes in MDA-MB 231 and SUM 149 cells compared to MCF7 cells was observed. There were no significant differences in centrosome volume between the MDA-MB-231 and SUM 149 cells.

Centrosome amplification is a hallmark of cancer; however, supernumerary centrosomes lead to multipolar spindle formation which is not compatible with survival. To overcome this issue centrosomes form clusters that assemble into a pseudo-bipolar spindle for the cell to go through mitosis. In our study we measured centrosome amplification and clustering in untreated cells from all the cell lines. Figure 3A show cells with amplified centrosomes (> 2 centrosomes) while Figure 3B shows interphase and metaphase cells with clustered centrosomes. We determined the percentage of cells with clusters and measured the centrosome volume using the NIS-Elements software. Figure 3C shows the extent of centrosome clustering in untreated cells from each of the 4 cell lines. Interphase clustering was significantly higher (*P* < 0.05) in MCF7, MDA-MB 231 and SUM 149 as compared to MCF-10A (2.64-fold, 3.54-fold and 5.05-fold increases, respectively). Comparing the basal to the luminal cells, we observed a significant (*P* < 0.05) 34% increase in interphase clustering between MDA-MB 231 and MCF7 and between SUM 149 and MCF7 (91% increase). In addition, SUM 149 interphase clustering was significantly higher than the observed MDA-MB 21 clustering (43% increase). Metaphase clustering was also significantly higher (*P* < 0.05) in MCF7, MDA-MB 231 and SUM 149 as compared to MCF-10A (1.9-fold, 3.41-fold and 4.92-fold increases, respectively). Comparing the basal to the luminal cell lines, we observed a significant (*P* < 0.05) 1.8-fold increase in metaphase clustering between MDA-MB 231 and MCF7 and 2.6-fold increase between SUM 149 and MCF7. In addition, SUM 149 metaphase clustering was 1.44-fold significantly higher (*P* < 0.05) than the observed MDA-MB 231 clustering.

Figure 3D shows the differences in centrosome volumes in the different cell lines. Centrosome volume was significantly higher (*P* < 0.05) in MCF7, MDA-MB 231 and SUM 149 as compared to MCF-10A (47%; 241% and 221%, respectively). Comparing the basal to the luminal cell lines, we also observed a significant increase (*P* < 0.05) in centrosome volumes in MDA-MB 231 (63% increase) and SUM 149 (50% increase) cells compared to MCF7 cells. There were no significant differences in centrosome volume between the MDA-MB-231 and SUM 149 cells.

### 2-ME induces centrosome declustering

**Figure 4:**
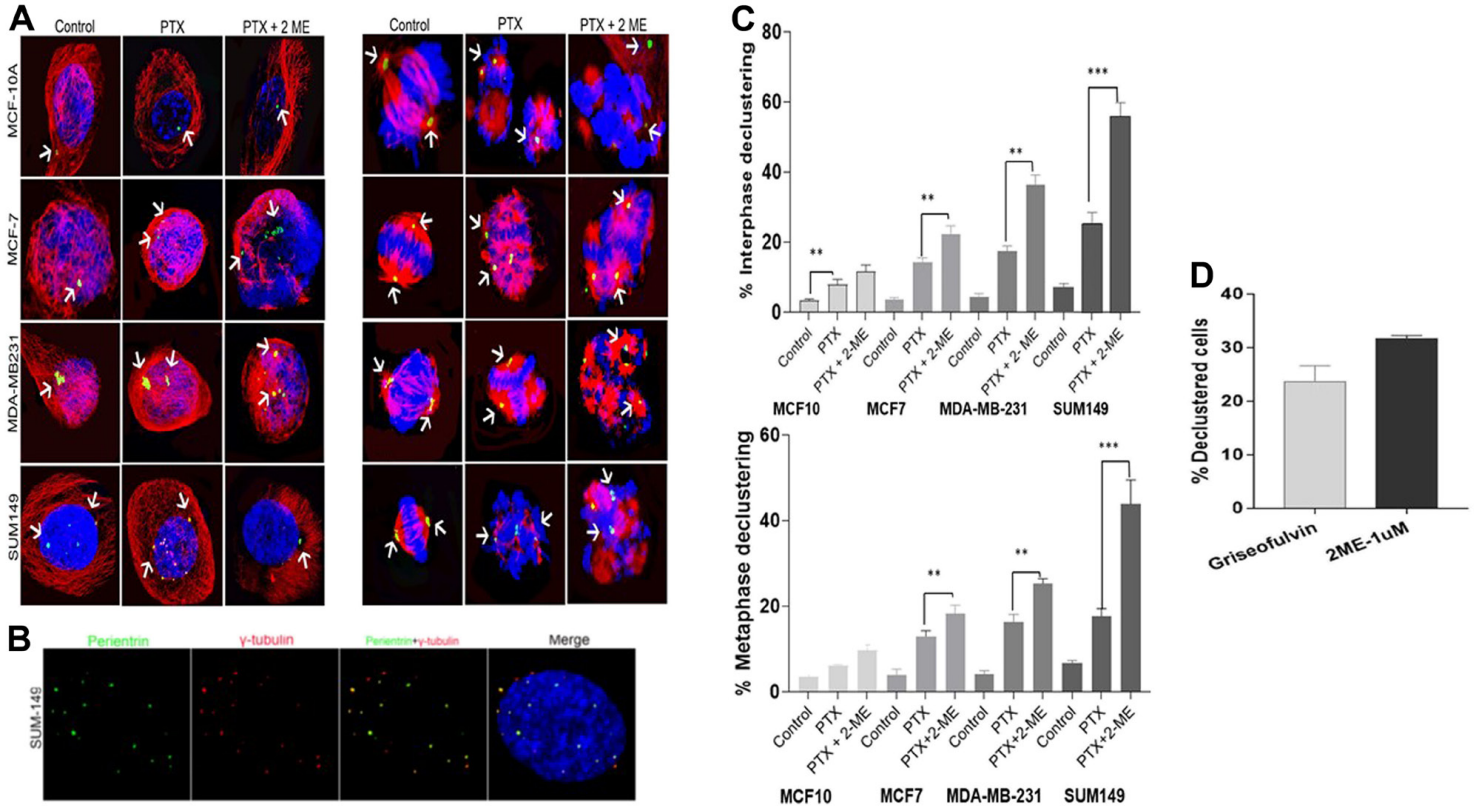
Centrosome declustering using gamma-tubulin to differentiate between centrosome declustering and centrosome fragmentation. (**A**) Centrosome declustering in interphase and metaphase cells in the different cell lines. (**B**) Shows the association of gamma-tubulin with the intact centrosomes and not fragmented ones. (**C**) Treatment with both PTX and 2-ME induced a significantly higher interphase declustering in cancer cells compared to PTX alone (56% increase for MCF7, 208% for MDA-MB231 and 218% for SUM149, respectively). Treatment with both PTX and 2-ME induced a increases in metaphase declustering in cancer cells compared to PTX alone (1.4-fold, 1.56-fold and 2.48-fold increase for MCF7, MDA-MB231 and SUM149, respectively. (**D**) 1 uM 2-ME induces similar declustering effect as the declustering drug Griesofulvin confirming the centrosome declustering effect of 2-ME.

Centrosome declustering was assessed in both interphase and metaphase stage of the cell cycle in all cell lines (Figure 4A). We used gamma-tubulin to differentiate between centrosome declustering and centrosome fragmentation. Gamma-tubulin is associated with intact centrosomes and not fragmented ones (Figure 4B). Declustering was observed in both interphase and metaphase cells (Figure 4C) indicate that treatment with both PTX and 2-ME induced a significant higher (*P* < 0.05) interphase declustering in cancer cells compared to PTX alone (56% increase for MCF7, 208% for MDA-MB231 and 218% for SUM149, respectively).

Comparing the basal to the luminal cell lines, we also observed that treatment with both PTX and 2-ME leads to a significant increase (*P* < 0.05) in interphase declustering. A 1.64-fold and 2.5-fold increase was observed in MDA-MB 231 and SUM 149 compared to MCF7 cells, respectively). In addition, a significant 1.53-fold increase (*P* < 0.05) in interphase declustering was observed in SUM149 compared to MDA-MB-23.

Similarly, treatment with both PTX and 2-ME induced significant (*P* < 0.05) increases in metaphase declustering in cancer cells compared to PTX alone (1.4-fold, 1.56-fold and 2.48-fold increase for MCF7, MDA-MB231 and SUM149, respectively; Figure 4C). Comparing the basal to the luminal cell lines, we observed that treatment with both PTX and 2-ME leads to a 1.4-fold increase (*P* < 0.05) in metaphase declustering in MDA-MB 231 and a 2.4-fold increase in SUM 149 compared to MCF7 cells. In addition, we observed a 1.7-fold significant increase (*P* < 0.05) in metaphase declustering in SUM 149 compared to MCF7 cells.

To confirm the declustering effect of 2-ME, we exposed SUM 149 cells to 50 uM Griseofulvin, a known centrosome declustering agent. Figure 4D shows that 1 uM 2-ME induced similar declustering effect as Griesofulvin. While treatment with Griesofulvin induced 23% declustered cells, treatment of cells with 2-ME induced 33% declustered cells, confirming the centrosome declustering effect of 2-ME.

## DISCUSSION

The naturally occurring estrogen metabolite 2-ME, possesses antiangiogenic and proapoptotic activity [22]. It has clinical benefit in treatment of cancer and multiple *in vitro* and *in vivo* studies reported that 2-ME to induces cell death and inhibits angiogenesis across many tumor types [23–26]. In a recent study, Massaro et al. [27] showed that 2-ME has antiproliferative and cytotoxic effects in 2D and 3D human melanoma cell culture models. 2-ME does not possess estrogenic activity due to its week affinity to estrogen receptors, which help avoid causing estrogen-dependent diseases such as breast cancer and makes it a promising anticancer agent for this disease [28]. 2-ME was used in Phase I/II clinical trials for single drug treatment of several cancer including glioblastoma, prostate, multiple myeloma, renal and breast cancers and was associated with few side effects [29]. Overall, toxicity was low and there was evidence of efficacy [30, 31]. Consistent with our findings, 2-ME has been reported to enhance the therapeutic effect of microtubule-disrupting agents such as taxanes [31]. Despite the reported beneficial antitumor effects of 2-ME, the exact mechanisms of 2-ME induced cytotoxicity in breast cancer remains unclear. Here we show that 2-ME reduces paclitaxel toxicity by significantly increasing spindle apparatus abnormalities.

In our study, cancer cells treated with PTX showed significant mitotic spindle defects in the form of chromosome misalignment as evident by lagging chromosomes in metaphase and anaphase and formation of bridges as well as increase in multipolar cell as compared to untreated cells. This mechanism of action of PTX is consistent with previous reports showing that PTX efficacy is primarily due to chromosome missegregation [32]. Our data indicate that concomitant treatment with 2-ME leads to a significant increase in the metaphase misalignment in all breast cancer cells evaluated. Similarly, cells treated with 2-ME and PTX showed a significant increase in the number of multipolar cells in MCF7, MDA-MB-231 and SUM 149, thus confirming our hypothesis that 2-ME sensitizes the cells to the cytotoxic effects of PTX. These data are consistent with previous studies indicating that the naturally occurring estrogen metabolite 2-ME is a multitarget drug with multiple mechanisms of action, including antiproliferative, antiangiogenic and proapoptotic activity [22].

The major proposed mechanism of 2-ME is through inhibiting polymerization of tubulin thus disrupting the microtubule function [33], leading to activation of spindle assembly checkpoints causing metaphase arrest. Here, we show centrosome declustering as a novel mechanism by which 2-ME induces metaphase arrest and subsequent cell death. Our data indicate that 2-ME targets cells with clustered supernumerary centrosomes, in interphase and mitotic phases, declustering them, generating multipolar cells that lead to cell cycle arrest.

Centrosome amplification is a hallmark of cancer and is observed in about 75% of breast tumors including premalignant lesions. It is associated with high grade in DCIS and invasive breast cancer [34]. Centrosome amplification promotes invasive tumor behavior and enhanced migratory ability of cancer cells [35]. Cells with supernumerary centrosomes (Figure 3A) are not compatible with cell survival and therefore the cells cluster centrosomes (Figure 3B) into two polar groups allowing the bipolar division to occur. Such centrosome clustering promotes further chromosome missegregation which drives tumor evolution [36]. Our data show that untreated MCF7, MDA-MB-231 and SUM 149 cancer cells had a significantly higher number of cells with centrosome clustering in both metaphase and interphase as compared to normal MFC10A cells. SUM 149 harbored the most cells with centrosome clusters (5.05-fold and 4.92-fold increases in interphase and metaphase cells, respectively) followed by MDA-MD231 (3.54-fold and 3.41-fold increases) and MCF7 (2.64-fold and 1.9-fold increases) (Figure 3C). Our data are consistent with recent studies reporting an association between amplified centrosomes and tumor aggressiveness in triple negative breast cancer [26] and aggressive pancreatic cancer [37].

A major finding in our study is the observation of significantly increased centrosome declustering in cancer cells treated with both PTX and 2-ME compared to cells treated with PTX alone. Centrosome declustering kills cancer cells with supernumerary centrosomes while sparing normal cells [38]. Studies have shown that unlike *in vitro* cell cultures, cancer cells in tumor tissues have low mitotic indices and proliferative rates [39] explaining why classical anti-mitotic drugs failed to translate their preclinical efficacy into clinical response in clinical trials [40]. It has been suggested that microtubule targeting agents should target interphase cells in order act on a large proportion of tumor cells [41]. Interestingly, our results showed that while concomitant treatment of cancer cells with both PTX and 2-ME was significantly more effective than PTX alone, the effect was more pronounced in interphase cells suggesting that 2-ME may be a strong declustering agent. This was further confirmed when we compared the declustering effect of 2-ME alone versus Griseofulvin.

In conclusion, to our knowledge this is the first report documenting centrosome declustering as a mechanism of action for anticancer effect of the naturally occurring 2-ME. Such finding may have significant therapeutic implications as it can provide a promising treatment strategy for cancer patients where tumor cells could be therapeutically targeted more efficiently. 2-ME targets cancer cells which possess supernumerary centrosomes, thus allowing the use of lower concentrations of taxanes, reducing unwanted side effects and reducing tumor resistance to these therapeutics.

## MATERIALS AND METHODS

### Cell lines and culture

Luminal A: MCF-7 (ER+, PR+/−, HER2-); Basal: MDA-MB-231 (ER-, PR-, HER2-) and SUM-149 (inflammatory breast cancer) BC cell lines from ATCC^®^ (Manassas, VA) were cultured per vendor recommendations. The MCF10A normal breast cell line was used as the non-cancer control. Briefly, cells were cultured in BEGM medium (Lonza^®^, Alpharetta, GA) supplemented with 0.5 ng/ml human recombinant epidermal growth factor (0.5 ng/ml), bovine pituitary extract (50 μg/ml), hydrocortisone (0.5 μg/ml), insulin (5 μg/ml), transferrin (10 μg/ml), epinephrine (0.5 μg/ml) and retinoic acid (0.1 ng/ml), in addition to standard penicillin (100 u/ml) and streptomycin (100 μg/ml) and 5% CO_2_ at 37°C until reaching 80% confluency. Authentication of the cell lines was performed using a short tandem repeat analysis.

### Paclitaxel (PTX) and 2-ME treatments

PTX (Sigma, St. Louis, MO) was dissolved using DMSO (0.1% of final volume) and a stock solution of 10 μM was prepared in cell culture medium. Cells were seeded into 96-well plates at a density of 1 × 10^4^ cells/well and were treated for 72 hours with 8 different PTX concentrations (1–128 nM). Cytotoxicity was assessed at the end of drug exposure using the XTT assay from ATCC^®^ (ATCC Cat # 30–1011K). The IC_50_, the drug concentration at which 50% growth inhibition is achieved, was calculated using Sigma Plot software. Experiments were repeated in presence of 1 μM 2-ME (Sigma^®^) dissolved in DMSO and the IC_50_ was again determined as described. This 2-ME concentration was based on the literature [42] and on preliminary experiments we conducted with different 2-ME concentrations (data not shown). The optical density was measured at 490 nm with a reference wavelength at 650 nm in a microplate reader (Biotech^®^, plate reader). All experiments were performed in triplicate and in low-light conditions.

### Immunofluorescence and antibodies for detection of multipolar spindle and centrosome amplification

Immunofluorescence (IF) was performed following standard protocols detailed elsewhere [43, 44]. Spindle staining was performed in combination with centrosome staining with a monoclonal antibody to α-tubulin (1:1,000, Sigma #T6074) in addition to the rabbit polycolonal anti-pericentrin antibody (Abcam-ab4448, Cambridge, UK). Secondary antibodies were Alexa Fluor 488 or 555 (Invitrogen) at a dilution of 1:500 and incubated for 1 h at room temperature. Nuclear staining was performed by incubating cells with DAPI (4′, 6-diamidino-2-phenylindole) containing mounting media (Vectashield, Burlingame, CA, USA). Approximately 30 spindles/sample were examined. Asymmetric, tri-, tetra-, or multipolar spindle patterns were considered aberrant [45]. The percentage of multipolar spindles was determined as those that contained more than two spindle poles. The additional spindle poles were counted by pericentrin staining in 100 metaphase cells/ concentration per time.

### Centrosome amplifications and clustering

We used standard protocols for centrosome staining [25]. We used a primary antibody to pericentrin (Covance^®^, Princeton, NJ, USA), followed by a Cy3-conjugated secondary antibody. This process allows the identification of abnormal centrosome size (diameter at least twice that of centrosomes) and abnormal centrosome count (> 2 centrosomes present for treated vs. untreated control cells) in 100 cells/condition. Mitotic cells with engaged centrioles have 2 centrosomes with 2 centrioles each, whereas cells with evidence of amplification either have 3 centrosomes or 4 centrosomes each with a single centriole. In addition, centrosome cluster volume was assessed by measuring the defining region of interest around z-stack images of 25 randomly-selected interphase and metaphase clusters. Cluster volume is an indication of the severity of the clustering, showing how abnormal the number/volume of the centrosomes in a given sample [46]. The volume of the cluster was calculated using the NIS-Elements AR 5.11 software by Nikon^®^. Extent of centrosome declustering was recorded as the number of cells with declustered centrosomes as well as the number of declustered centrosomes/cell in each experimental condition. Differentiating between centrosome declustering and centrosome fragmentation was done by staining of gamma-tubulin which is associated with intact centrosomes but not fragmented ones. The percentage of cells with both pericentrin and gamma-tubulin or pericentrin alone was calculated. The rate of centrosome alterations was compared between the different BC subtypes.

### Confocal imaging, image acquisition and analysis

Fluorescence image acquisition was performed using a Nikon A1R confocal imaging system controlled by the Nikon NIS Elements software (Nikon, Japan). The objective lens was an oil immersion Plan-Apo _60 numerical aperture (NA) 1.40 lens (Nikon). Images were acquired as Z-stacks at 0.2-mm intervals and maximum-intensity projections were generated using the NIS Elements software (Nikon). Confocal *z* stacks were acquired with sections were 0.5 μm. Image acquisition settings were kept constant throughout the experiments.

### Statistical analysis

Data presented as Mean ± Standard Error was analyzed using the parametric analysis of variance (ANOVA) and independent Student’s *t*-tests corrected for the numbers of comparisons via Bonferroni correction to compare between differences in the outcome variables between cell lines. *P*-value < 0.05 were considered significant.

## Abbreviations

E2: estrogens
2-ME: 2-Methoxyestradiole
BC: breast cancer
PTX: paclitaxel
IC_50_: half maximal inhibitory concentration
ER: estrogen receptor
PR: progesterone receptor
HER2: human epidermal growth factor receptor 2
4-OHE: 4-hydroxyestradiol
